# α-Linolenic acid induces clearance of Tau seeds *via* Actin-remodeling in Microglia

**DOI:** 10.1186/s43556-021-00028-1

**Published:** 2021-02-09

**Authors:** Smita Eknath Desale, Subashchandrabose Chinnathambi

**Affiliations:** 1 Neurobiology Group, Division of Biochemical Sciences, CSIR-National Chemical Laboratory, Dr. Homi Bhabha Road, Pune, 411008, India; 2 Academy of Scientific and Innovative Research (AcSIR), Ghaziabad, 201002, India

**Keywords:** α- Linolenic acid, Lamellipodia, Filopodia, Membrane ruffling, Arp2/3 complex, Phagocytosis, Tauopathy

## Abstract

Alzheimer’s disease (AD) is known by characteristic features, extracellular burden of amyloid-β and intracellular neuronal Tau. Microglia, the innate immune cell of the brain has the ability to clear the burden of accumulated proteins *via* phagocytosis. But the excessive proinflammatory cytokine production, altered cellular signaling and actin remodeling hampers the process of migration and phagocytosis by microglia. Actin remodeling is necessary to initiate the chemotactic migration of microglia towards the target and engulf it. The formation of lamellipodia, filopodia, membrane ruffling and rapid turnover of F-actin is necessary to sense the extracellular target by the cells. Omega-3 fatty acids, are known to impose anti-inflammatory phenotype of microglia by enhancing its ability for migration and phagocytosis. But the role of omega-3 fatty acids in cellular actin remodeling, which is the basis of cellular functions such as migration and phagocytosis, is not well understood. Here, we have focused on the effect of dietary supplement of α-linolenic acid (ALA) on extracellular Tau internalization and assisted actin polymerization for the process. ALA is found to induce membrane ruffling and phagocytic cup formation along with cytoskeletal rearrangement. ALA also enhances the localization of Arp2/3 complex at the leading edge and its colocalization with F-actin to induce the actin polymerization. The excessive actin polymerization might help the cell to protrude forward and perform its migration. The results suggest that dietary supplement of ALA could play a neuroprotective role and slow down the AD pathology.

**Supplementary Information:**

The online version contains supplementary material available at 10.1186/s43556-021-00028-1.

## Introduction

Alzheimer’s disease (AD) is characterized by accumulation of extracellular amyloid-β plaques and intracellular deposition of neurofibrillary tangles (NFTs), occurred due to post-translational modification in Tau protein [1]. Tauopathy is characterized by excessive abnormal phosphorylation of Tau, which reduces its affinity towards microtubules that leads to instability in microtubule functions and loss of synaptic neurons [1–3]. Various studies have reported the ability of Tau aggregates to spread in a prion-like manner in neuronal culture through various cellular uptake mechanisms to demonstrate template-dependent aggregation of intracellular monomeric Tau [4–6]. The extracellular species of Tau can exert toxicity in cultured hippocampal neurons *via* unbalanced calcium metabolism and causes cell death [7]. Microglia, the obligate phagocytes of the brain are capable of uptaking the bioactive Tau seed and neutralize it through degradation [8, 9]. However, Microglia can contribute to Tau pathology by releasing the toxic Tau seed into the media due to its inefficiency in processing of Tau [10]. The relationship between Tau pathology and neuroinflammation by microglia is well-known, that reflects the inability of microglia to fully process the Tau seed.

Physiologically, microglia in resting state inspects the brain environment with their long extensions. The communication with other glia cells and neuron takes place* via* various cellular receptors, chemokine signals and soluble factors [11, 12]. After sensing the tissue injury, pathogen invasion or plaque deposition; microglia becomes a motile effector cell and acquire an amoeboid form that can migrate towards a concentration gradient of chemotactic signals. For the 2-dimensional migratory motion of microglia, the cells are supported by protruding fan-shaped actin-based structures (lamellipodia) on leading edge and F-actin-rich thin extensions (filopodia) on the rear edge [13, 14]. In this migratory state, microglia manifest the presence of podosomes, which are actin-rich structures that mediates adhesion to substratum, migration and ectracellular matrix (ECM) degradation for invasion [15]. Microglial migration followed by Ca^2+^ regulation is necessary for phagocytosis, and the phagocytic activity is diminished in Stromal interaction molecule (STIM) depleted microglia [16]. The initiation of signaling cascades regulating the actin cytoskeleton, which involves membrane protrusions and ruffling to increase the surface areas, are obligatory for the phagocytosis [17]. The polarized state of microglia is maintained by the cytoskeletal network, wherein actin provides directional sensing while microtubule dynamics provide mechanical strength for the forward movement of cells [18]. The co-ordinated polymerization of actin filaments provide a protrusive force to the cell for forward movement. Actin related protein 2/3 complex (Arp2/3) along with other actin-binding proteins play an essential role in nucleating the branching of actin filaments [19]. Ionized calcium-binding adaptor molecule-1 (Iba-1), a specific marker of microglia/macrophages lineage, is also reported to have a key role in the function of activated microglia [20, 21]. In the process of membrane ruffling and phagocytic cup formation along with actin network, Iba-1 protein of microglia plays a pivotal role.

In AD, ability of microglia to carry out phagocytosis of extracellular Aβ plaques as well as aggregated Tau seeds are most affected along with the disrupted actin cytoskeleton [22]. The disrupted actin cytoskeleton due to Aβ *via* Aβ-mediated dysregulation of phosphoinositides signaling could be consider as a cause of pathology [23]. The extracellular Tau oligomer and aggregates have the potential to remodulate the actin cytoskeleton for active phagocytosis [24]. Polyunsaturated fatty acids (PUFAs), majorly omega-3 fatty acids have a beneficial role in the brain, which improve cognitive function and memory loss. PUFAs exist in the form of phospholipid content in the cell membrane of the brain cells, which efficiently influences the membrane receptor expression, signaling cascade and effector functions of the cell [25–27]. Omega-3 fatty acids, especially DHA and EPA exerts anti-inflammatory phenotype in the brain immune cells by modulating the lipid composition of cell membrane and cell surface receptor expression [28–30]. The cellular functions such as phagocytosis, migration are carried out with the assistance of actin remodeling [28, 31]. For the process of phagocytosis to engulf extracellular pathogens, membrane extension-induced by actin remodeling and phosphoinositides induced-signaling is imperative [32, 33]. Omega-3 PUFAs are known to significantly alter cytoskeleton-associated gene expression including Ras-related C3 botulinum toxin substrate 1 (Rac1), Cell division control protein 42 homolog (CDC42), Wiskott–Aldrich Syndrome *protein* (WASP) and Arp2/3 complex [28].

In this study, we aim to understand the effect of α-Linolenic acid, a precursor of DHA and EPA, on actin remodeling related to phagocytosis and migration. In Tauopathies, extracellular Tau seeds tend to activate microglia to induce inflammatory activation. However, the study involves understanding the effect of ALA on phagocytosis and related actin-remodeling in microglia in the presence of extracellular Tau seeds. We have further studied the role of Iba-1 and Arp2/3 complex in membrane ruffling and actin polymerization. We observed the efficiency of ALA to induce actin remodeling necessary for phagocytosis.

## Results

### Internalization of extracellular Tau-induced by ALA

**Fig. 1:**
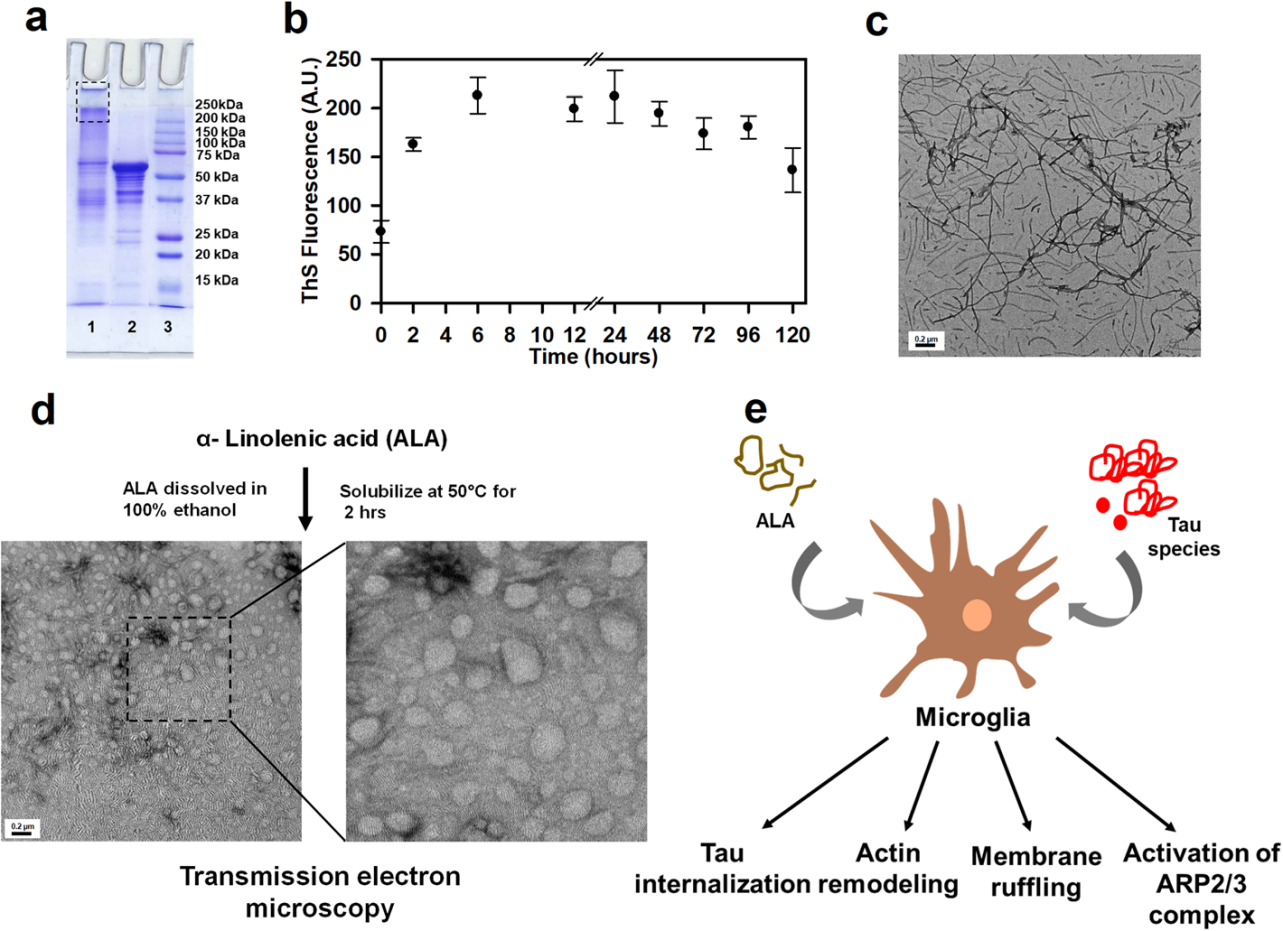
Characterization of hTau40 aggregates and experimental outline. **a** Human Tau40 aggregates were prepared for cellular studies. Tau aggregation was carried out in presence of heparin for 120 h. Higher-order aggregates have been characterized by SDS-PAGE analysis. The protein band for higher-order aggregates were visible above 250 kDa (lane 1), the protein band corresponding to monomer is indicated (lane 2). **b** Tau aggregates were studied by ThS fluorescence kinetics for 120 h. **c** Tau aggregates were visualized *via* transmission electron microscopy (TEM) to understand Tau fibrils formation, scale bar is 0.2 μm. **d** ALA was prepared as a starting material in the presence of 100% ethanol and solubilized at 50 °C for 2 h. The transmission electron microscope image indicates the morphology of ALA, scale bar is 200 nm. **e** Schematic representation of experimental proceeding depicts the role of ALA in phagocytosis of extracellular Tau and necessary actin remodeling for the process

**Fig. 2:**
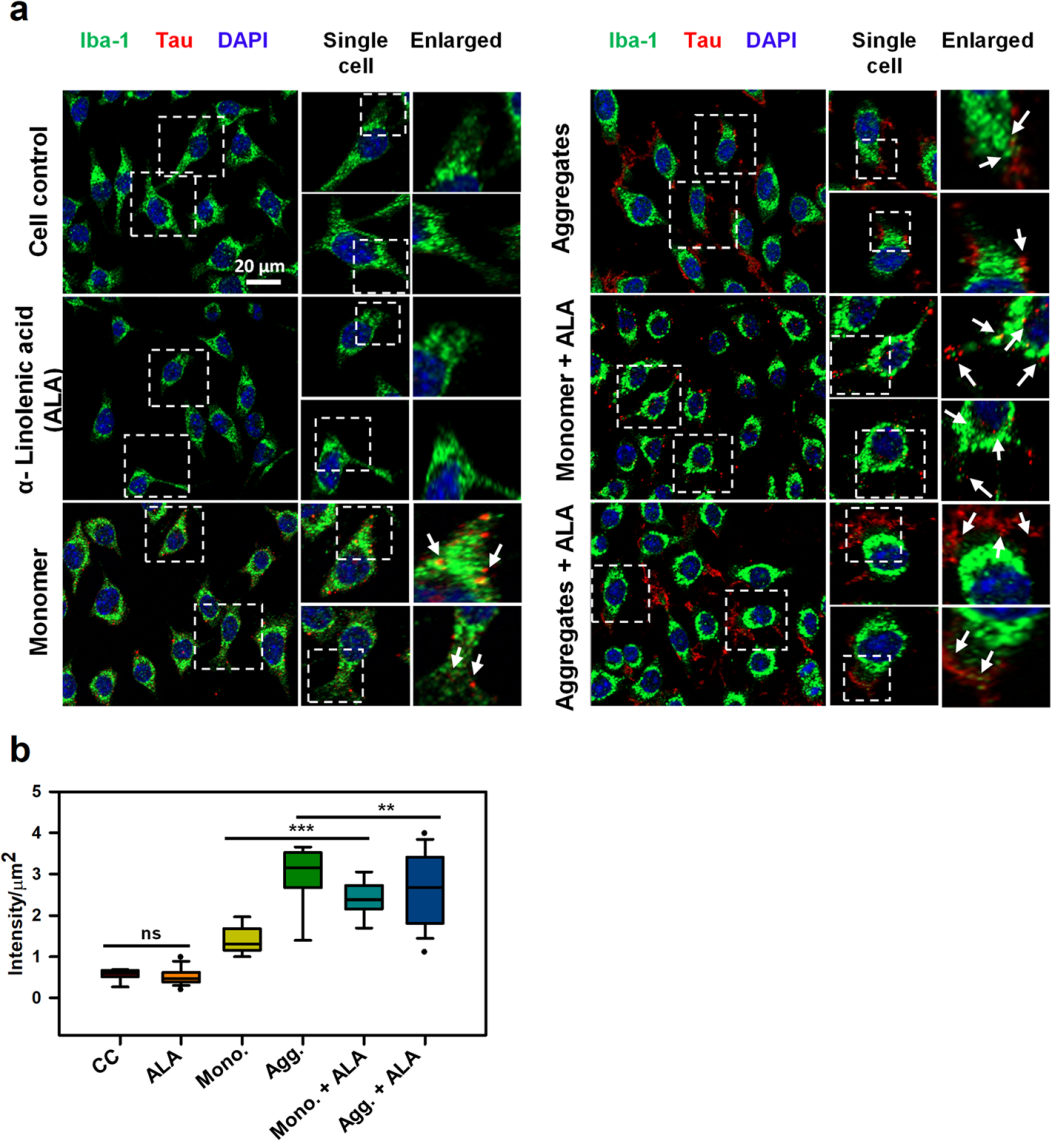
Internalization of extracellular Tau in Microglia. **a** Study of internalization of extracellular Tau monomer and aggregates in the presence of ALA. The treatment was given for 24 h and studied with fluorescence microscopy to understand the internalization of Tau (red) in Iba-1 (green) positive cells. Figure showing fluorescence microscope image, single cell and enlarged panel indicates that the internalized Tau denoted with white arrow marks. The enlarged region is highlighted with white dotted square that indicates internalized area in the cells. Axio observer 7.0 Apotome 2.0 Zeiss microscope was used to the study of internalization extracellular Tau. **b** Quantification of internalized Tau was carried out with a total intensity of Tau (red) per μm^2^ area inside the cell was analyzed by ZEN 2.3 software. The significance was analyzed with Tukey’s Kramer, significant when mean difference between treatment groups (X-X’) > T (Tukey’s criteria). The significance of conditions of Tau monomer and aggregtes with ALA is *p* < 0.005. annotations used in graph are as follows- CC (Cell control), ALA (α-linolenic acid), Mono. (monomer), Agg. (aggregates), Mono. + ALA (monomer plus ALA), Agg. + ALA (aggregates plus ALA)

Microglia are professional phagocytes of the brain, which assist in clearing apoptotic cells, axonal and myelin debris, central infection of bacteria and viruses, misfolded aggregated protein-like Aβ and participate in synaptic pruning [34]. Along with Aβ plaques, microglia also recognizes extracellular Tau oligomers and aggregates and undergo activation to engulf the Tau seeds. However, the increased production of IFN-γ and TNF-α by microglia hampers its phagocytic ability and are toxic to neuronal/microglial cells [22]. PUFAs treatment reduces the production of inflammatory cytokines while exerts anti-inflammatory cytokines production as well as improves phagocytosis [29]. We prepared human Tau40 (hTau40) aggregates in vitro in the presence of heparin as polyanionic agent, which can induce Tau aggregation [35]. The hTau40 aggregates were further characterized for their higher molecular weight aggregates by SDS-PAGE (Fig. 1a). The propensity of Tau aggregation was studied with ThS fluorophore that binds to Tau aggregates and gives the extent of aggregation (Fig. 1b). The fully formed mature Tau fibrils were assessed with transmission electron microscopy (TEM) (Fig. 1c). ALA was prepared by dissolving in 100% ethanol, followed by heating at 50 °C for 2 h and are characterized by TEM analysis (Fig. 1d). The scheme represents the experimental proceeding of the study as Tau internalization and the changes in actin cytoskeleton associated with the process (Fig. 1e). To study the ability of ALA to cause anti-inflammatory effects in microglia, we observed the phagocytosis of extracellular Tau. The N9 cells were treated with 1 μM Tau monomer, 1 μM Tau aggregates along with 40 μM ALA as a test, and respective control of Tau monomer, aggregates, ALA and cell control (untreated) were kept for comparion. Internalization of extracellular Tau in microglia was studied with fluorescence microscopy. Tau (red), Iba-1 (green) were detected in microglia cells after 24 h exposure of Tau and ALA to N9 microglia culture (Fig. 2a) The intracellular localization of internalized Tau in microglia are indicated in fluorescence images (Fig. 2a). The single cell and enlarged panel indicate the zoomed area of microglia with the internalized Tau, marked with the white arrow marks (Fig. 2a). The phagocytic ability of microglia for Tau monomer as well as aggregates has been observed to increase upon exposure of ALA. The mean intensity of internalized Tau has been increased to 44% for Tau monomer upon ALA exposure, whereas, the increase of 49% has been observed for Tau aggregates (Fig. 2b). The statistical analysis suggests the significance difference between Tau and ALA treated groups as compared with control (untreated) and ALA groups. The internalization ability of microglia was found to be enhanced with ALA exposure.

### Effect of ALA on microglial polarization and activation

**Fig. 3:**
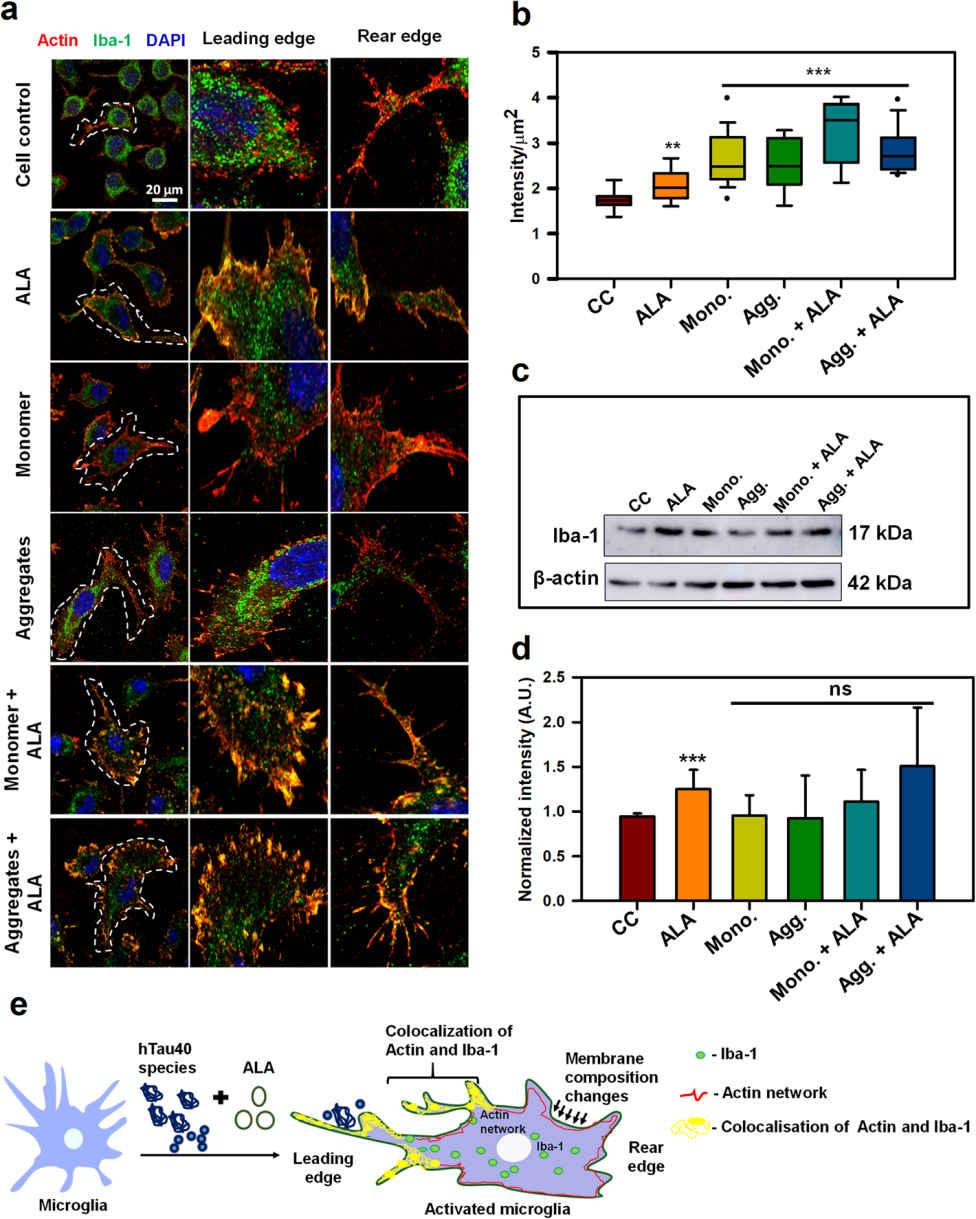
Modulation of actin network in activated microglia by ALA. Role of actin and Iba-1 in microglial migration and activation **a** N9 microglial cells were treated with Tau monomer, aggregates along with ALA for 24 h. After fixation cells were stained with β-actin (red) and anti-Iba-1 antibody (green). Colocalization of actin and Iba-1 was studied by Super-resolution confocal microscopy. The single cells are marked with white dotted lines in the merged image, scale bar is 20 μm. The migratory microglia are shown with the front; leading edge and rear edge and the colocalization of actin and Iba-1 in the edges assisting the microglia for membrane ruffling and phagocytosis. **b** The quantification of Iba-1 in cell indicating its activation status and its levels on increased phagocytosis. The increased levels of total intensity per μm^2^ area of microglia was calculated with ZEN 2.3 software, significance is (*p* < 0.001). **c** The protein expression of Iba-1 was measured by western blot analysis in all the treated groups after 24 h of treatment and stained with anti-Iba-1 and β-actin antibody for loading control. **d** Quantification of the intensity of protein bands from western blot normalized with the β-actin loading control. The conditions of Tau monomer and aggregtes with ALA are nonsignificant, (*p* > 0.05). **e** Schematic overview of actin remodeling at leading edge of microglia and involvement of Iba-1 in the process of phagocytosis after ALA treatment to cells. The significance was analyzed with Tukey’s Kramer, significant when mean difference between treatment groups (X-X’) > T (Tukey’s criteria). The annotations used in graph are as follows- CC (Cell control), ALA (α-linolenic acid), Mono. (monomer), Agg. (aggregates), Mono. + ALA (monomer plus ALA), Agg. + ALA (aggregates plus ALA).

The actin cytoskeleton is involved in membrane-associated remodeling, downstream signal transduction and provides a mechanical framework for phagosomes upon internalization. Actin cytoskeleton furnishes the platform for receptors and other signaling cascades. Co-ordinated cycles of actin polymerization and depolymerization occurs at phagosomes for its internalization and maturation. Membrane-ruffling and phagocytic cup formation is being assisted by actin cytoskeleton along with the Iba-1 protein in microglia. Iba-1 enhances membrane-ruffling and cross-linking of actin filaments, which assist to up-regulate the internalization events [20]. After interaction of extracellular target with phagocytic receptor, signal transduction associated with actin cytoskeleton is initiated in the cell. The necessary remodeling with the membrane-associated actin and introduction of more membrane protrusion enhance the process of phagocytosis [36]. The N9 microglial cells were treated with different groups that includes 1 μM hTau40 monomer, aggregates along with their respective treatment with 40 μM ALA and cell control (untreated), ALA control (only ALA treatment) was kept for the comparison. We studied the role of actin (red) and Iba-1 (green) in actin-remodeling and polarization of microglia on ALA exposure by immunofluorescence staining. In the activated migratory microglia, the polymerization of actin at leading edge is signified by the presence of fan-shaped lamellipodia and thin filamentous filopodia-like structure, whereas, long extension is observed at the rear edge (Fig. 3a). For the migratory cells such as immune cells, the front-edge protrusion of lamellipodia formed by the forces of actin polymerization indicates the migratory state of microglia since they sense the extracellular Tau species. Whereas; the finger-like protrusion called as filopodia would cause the slow migration in absence of lamellipodia [37]. The extracellular Tau can act as an activation factor for microglia. The ionized calcium-binding adaptor protein-1 (Iba-1), functions in actin-crosslinking in membrane ruffling, which plays a role during ramified to amoeboid change in microglia. This possibly relate the activation of microglia with increased expression of Iba-1 [38]. The immunofluorescence staining indicates enhanced colocalization between Iba-1 and actin at the leading edge of activated microglia cells in ALA treated groups (Fig. 3a). The expression of Iba-1 has found to be increased upon exposure of Tau as compared to cell control (untreated) that can notify the activation state of microglia (Fig. 3b, c, d). These results indicate the involvement of actin and Iba-1 in phagocytic cup formation and membrane ruffling (Fig. 3a, 4a) [20]. Intracellular intensity of Iba-1 was quantified *via* ZEN 2.3 software and it was found to increase significantly in both monomer and aggregates in presence of ALA. The statistical analysis suggest the significant difference among groups treated with Tau and ALA as compared with control (untreated) and ALA groups (*p* < 0.001) and the data was plotted as mean with the standard error between the individual conditions (Fig. 3b). Iba-1 levels were also quantified by western blot, Iba-1 levels have been increased with respect to cell control (untreatred), which is presented as the quantification with loading control (β-Actin) (Fig. 3c and d). The levels of Iba-1 although found to increase but data is found to be insignificant after post-hoc analysis. In this study, we observed that, exposure of ALA and Tau species to microglia triggers the microglial activation and enhance actin remodeling, which may induce processes such as migration and phagocytosis. Actin and Iba-1 play a major role in microglial migration and activation, which assist the phagocytosis (Fig. 3e).

**Fig. 4:**
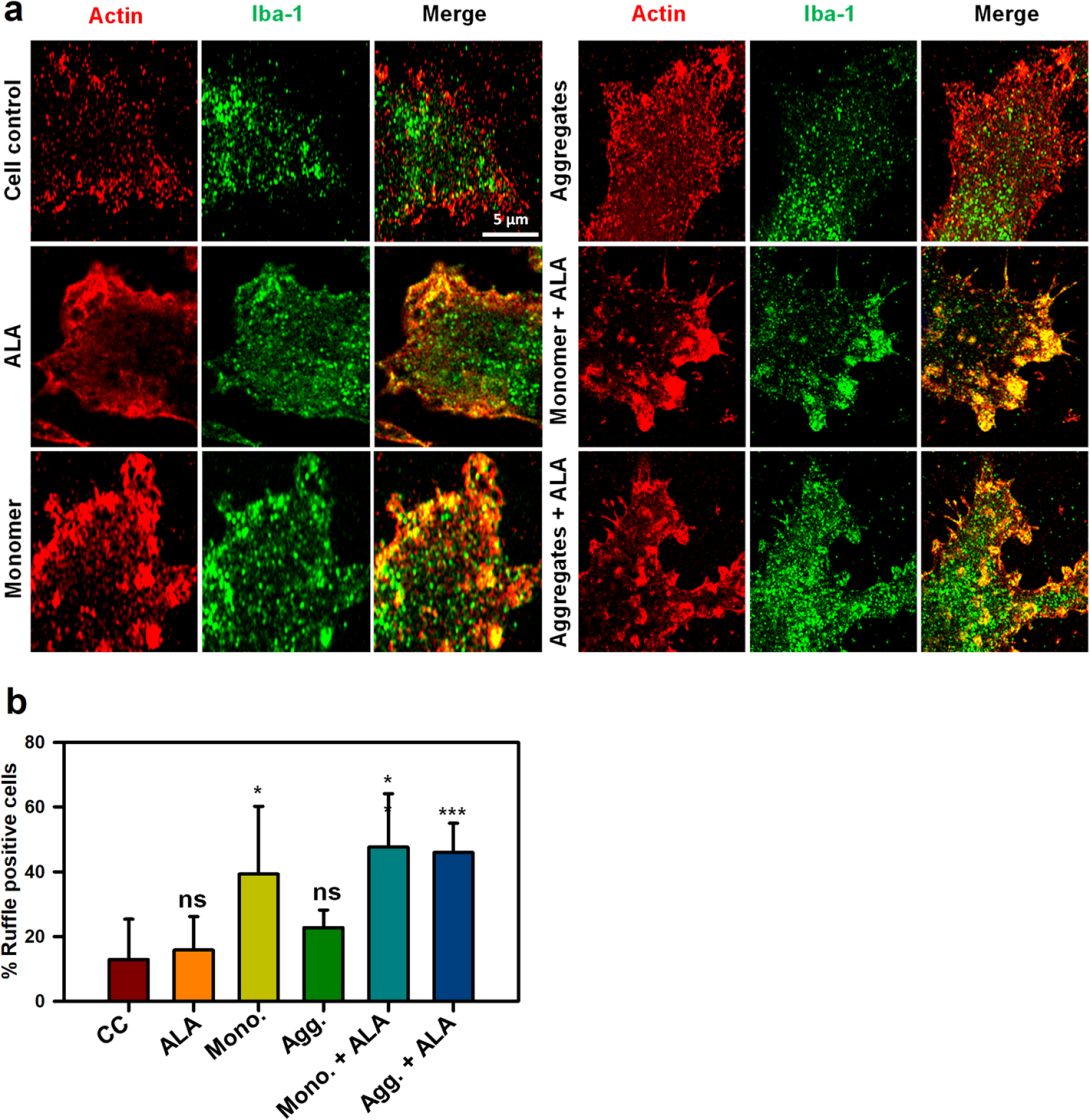
Formation of membrane ruffles induced by ALA. Role of actin and Iba-1 in the formation of membrane ruffling was studied upon exposure of ALA. The effect of ALA in membrane ruffling was observed with the super-resolution confocal microscopy. **a** N9 cells were treated with Tau monomer, aggregates and ALA for 24 h followed by staining with actin and Iba-1 to study the membrane ruffling. The zoom images are indicated with the colocalization of actin and Iba-1 showing membrane ruffling in images. The sacel bar is 5 μm. **b** The quantification of ruffle positive cells by measuring actin foci per cell were carried out for the percentage calculation. The conditions of Tau monomer and aggregtes with ALA are significant, (*p* < 0.005). The significance was analyzed with Tukey’s Kramer, significant when mean difference between treatment groups (X-X’) > T (Tukey’s criteria). The annotations used in graph are as follows- CC (Cell control), ALA (α-linolenic acid), Mono. (monomer), Agg. (aggregates), Mono. + ALA (monomer plus ALA), Agg. + ALA (aggregates plus ALA)

### Enhancement of membrane ruffling in presence of ALA

After the detection of the target, immune cells increase the membrane protrusion and membrane ruffling to increase the area in contact with the target to initiate the internalization process. Importance of actin polymerization and involvement of Iba-1 in membrane ruffling has been studied with Super-Resolution Confocal microscopy. The presence of ALA was found to enhance the membrane ruffling in both monomer and aggregates treated cells, which might enhance the chances of internalization in microglia, scale bar is 5 μm (Fig. 4a). The overall quantification of membrane ruffle positive cells were carried out with the number of actin foci per cell according to previous studies [39]. The quantification indicates the increased occurrence of membrane ruffle positive cells upon ALA exposure in both Tau monomer and aggregates treated cells. The statistical analysis suggest the significance difference amongst ALA and Tau treatment groups as compared to control (untreated) and ALA groups (*p* < 0.005) and the data was plotted as a mean with standard error between the conditions (Fig. 4b). The overall view of the zoomed images with separate panel is shown in supplementary figure, scale bar is 20 μm (Fig. S1). Respective cell have been marked with dotted line from where the enlarge areas have been observed.

### ALA enhance actin structure for migration and phagocytosis

**Fig. 5:**
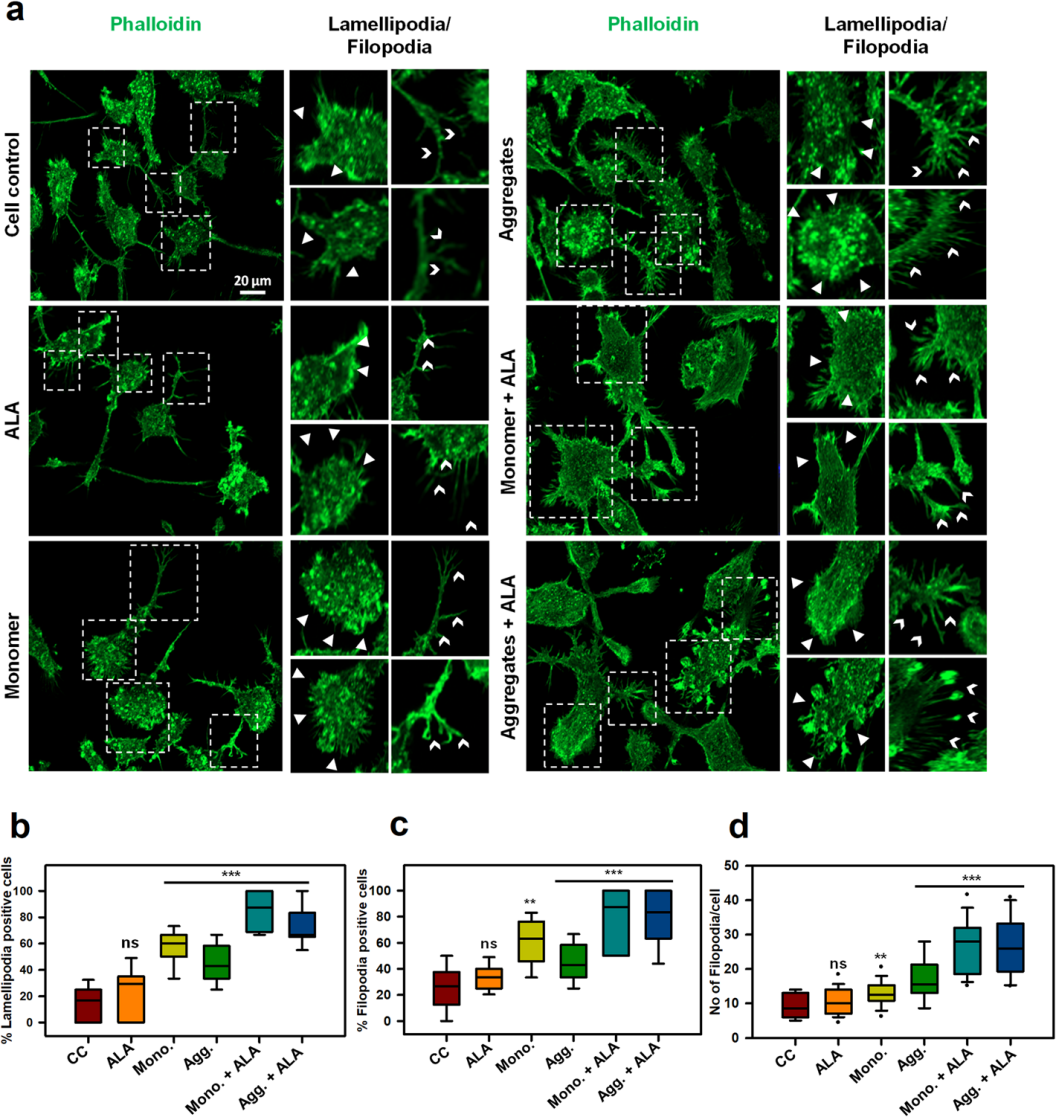
Enhancement of actin-rich structures for migration in microglia. Actin plays a significant role in the migration of microglia by providing mechanical strength as well as direction support. **a** Actin based structures lamellipodia, filopodia were observed after 24 h exposure Tau monomer, aggregates along with ALA to microglia cells by fluorescence microscopy. The enlarge panel indicates the presence of lamellipodia (white triangles), filopodia (white arrow heads) from the fluorescence image. The enlarged regions are denoted with the white dotted square in the image, scale bar is 20 μm. **b** The graph represents quantification of percentage of lamellipodia positive cells per 10 fields of different treatment groups, (*p* < 0.001) as compared to cell control (untreated) and ALA. **c** The graphical representation of percentage of filopodia positive cells per 10 fields of different treatment groups Tau monomer, aggregates along with ALA showed high significance of (*p* < 0.001) **d** Quantification of number of filopodia extensions present per cell per 10 fields of different groups, significance is (*p* < 0.001). The significance was analyzed with Tukey’s Kramer, significant when mean difference between treatment groups (X-X’) > T (Tukey’s criteria). The annotations used in graph are as follows- CC (Cell control), ALA (α-linolenic acid), Mono. (monomer), Agg. (aggregates), Mono. + ALA (monomer plus ALA), Agg. + ALA (aggregates plus ALA)

Immune cells are characterized by excessive motility to survey environment to find and destroy target pathogens. Actin polymerization provides necessary protrusion for the cells to move forward, which can be observed with the lamellipodia and filopodia to sense distant targets [19]. The actin-remodeling is important for phagocytosis, motility as well as matrix degradation [18]. We observed the role of actin-rich structures lamellipodia and filopodia in migration and phagocytosis. N9 cells were stained with F-actin (Phalloidin) to understand the fine actin structures (Fig. 5a). The sheet-like membrane protrusions at the leading edge and finger-like protrusions were visualized with phalloidin staining after 24 h exposure of Tau monomer, aggregates along with ALA. The lamellipodia-like front -edge protrusions have been increased upon Tau treatment along with ALA, which indicates ALA initiates the motility and phagocytosis of extracellular Tau (*p* < 0.001) (Fig. 5a, b). The data is significant as compared to control (untreated), ALA groups. The quantification of percentage lamellipodia positive cells have been performed on 10 different microscopic fields. The cells showing fan-shaped protrusion at the leading edge were considered as a lamellipodia positive cell [24, 40]. The actin spike, which are known as filopodia-like structures and the number of filopodia per cell have been increased in ALA exposed groups indicating activation and initiation of motility of microglia in the presence of ALA (*p* < 0.001) (Fig. 5a, c, d). The filopodia positive cells and number of filopodia per cells have been calculated as previous studies, any point protrude from the cell and more than 1 μm is considered as filopodia [24, 41]. The percentage filopodia positive cells have increased over ALA and Tau exposure of both monomer and aggregates, as compared to control groups (Fig. 5c). Statistical analysis suggests that, there is clear significant difference between Tau and ALA treated groups as compared with control (untreated), ALA groups. The number of filopodia per cell has increased significantly with ALA exposure in monomer and aggregates treatment, and the numbers of filopodia per cell are maximum in monomer with ALA treatment (*p* < 0.001) (Fig. 5d). The significance analysis indicated the significant difference between ALA and Tau treated groups with respect to control (untreated), ALA treated groups. Actin plays a major role in phagocytosis and migration, which can be observed by enhanced actin structures in the form of lamellipodia and filopodia upon ALA exposure. Therefore, the study suggested an independent beneficial effect of ALA as a omega-3 fatty acid on actin-remodeling.

### ALA enhances actin branching network

**Fig. 6:**
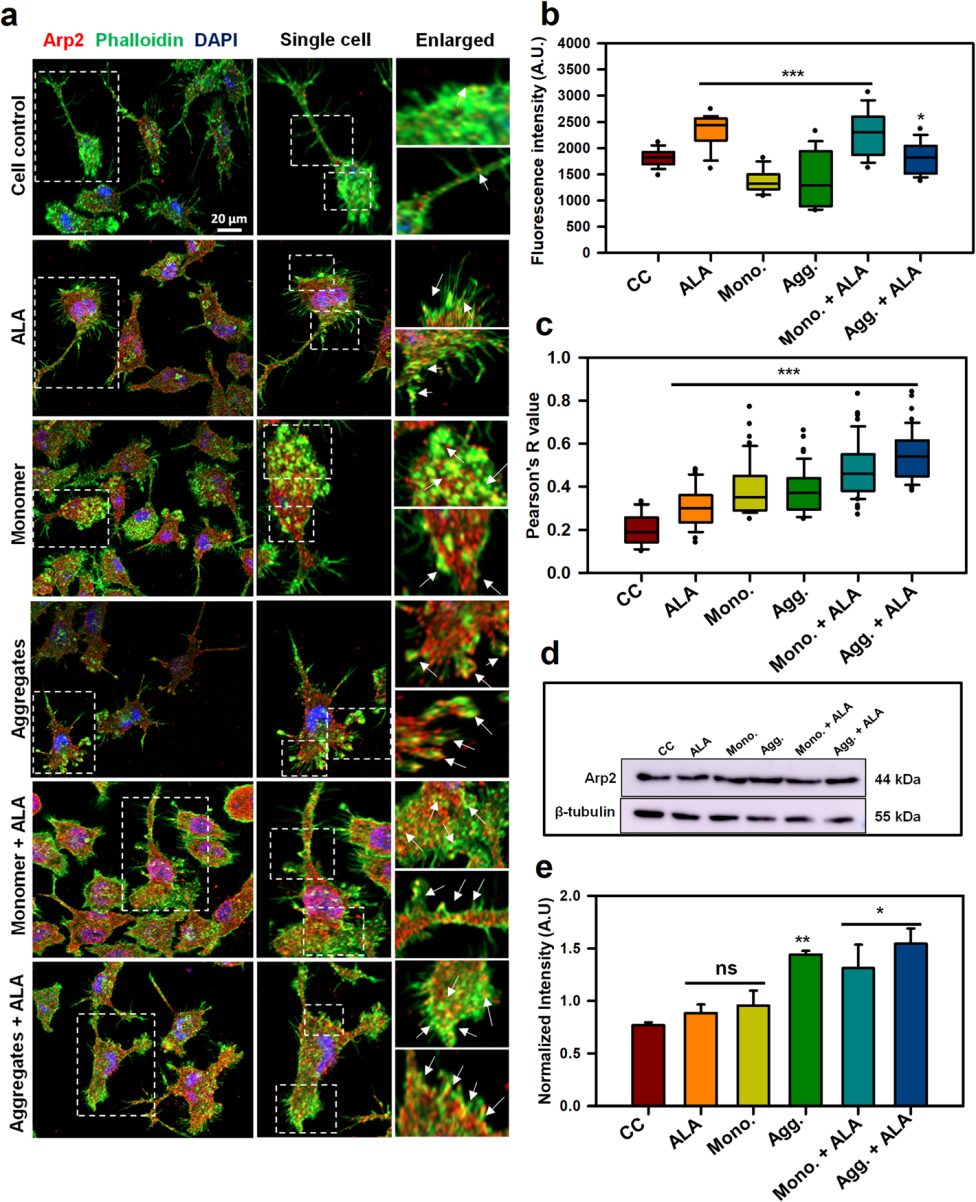
Enhancement of Arp2/3-mediated actin polymerization in the presence of ALA. Arp2/3 complex plays an important role in nucleating the actin polymerization and provide pushing force for the cell to move forward. After exposure of Tau monomer, aggregates and ALA the abundance of Arp2/3 in microglia was studied by fluorescence microscopy. **a** Immunofluorescence images indicate the microglia cells stained with Arp2/3 (red), phalloidin (green), DAPI, scale bar is 20 μm. The enlarged area indicates the colocalization of F-actin and Arp2/3 complex. The enlarged area has been taken from single cell panel and marked with white dotted squares. The white arrow marks indicates concentrated colocalized area inside the cell. **b** The intracellular intensity of Arp2/3 in the cell was quantified from immunofluorescence images with ZEN 2.3 software and plotted as a fluorescent intensity units (*p* < 0.05) for ALA treated groups as compared to cell control. **c** The Pearson’s R value for colocalization was calculated with ImageJ software. The conditions of Tau monomer and aggregtes with ALA are significant, (*p* < 0.001). **d** The quantification of protein levels of Arp2/3 was observed from western blot analysis after 24 h treatment of Tau monomer, aggregates and ALA. **e** The graph indicates quantification of western blot bands normalized with the loading control β-tubulin. The conditions of Tau monomer and aggregtes with ALA are significant, (*p* < 0.05). The significance was analyzed with Tukey’s Kramer, significant when mean difference between treatment groups (X-X’) > T (Tukey’s criteria). The annotations used in graph are as follows- CC (Cell control), ALA (α-linolenic acid), Mono. (monomer), Agg. (aggregates), Mono. + ALA (monomer plus ALA), Agg. + ALA (aggregates plus ALA)

The nucleation of actin filaments is required to start the assembly process of any actin-based structures. The protrusive edge of cell is especially enriched with orthogonally cross-linked actin filaments, which are induced due to the presence of assembly factors such as, Arp2/3 complex [42]. The protrusive force needed for the cell to move forward is provided by actin polymerization, where Arp2/3 complex plays a larger role to initiate the polymerization process. The increased density of Arp2/3 complex at the membrane of extending lamellipodia suggest the involvement of Arp2/3 complex in the formation of growing filaments [43]. We have studied the occurrence of Arp2/3 complex in microglia after treatment of Tau monomer, aggregates and ALA. The representation of images indicate Arp2/3 (red), phalloidin (green) and DAPI-nuclear staiming (blue) indicates the presence of Arp2/3 at the leading edge of cells, inducing more branching of actin filaments for the protrusive force (Fig. 6a). The single cell and enlarged panel indicates that colocalization of Arp2/3 complex with F-actin, and the degree of colocalization have found to increase with ALA exposure to cells. The degree of colocalization between F-actin and Arp2 have found to increased in test groups treated with Tau species and ALA, as compared to control groups (Fig. 6a). Figure S2 indicates the orthogonal section showing intracellular colocalization of F-actin and Arp2/3 in XY plane (Fig. S2). Intensity of Arp2/3 complex protein was calculated from immunofluorescence images to check the protein levels. The fluorescence intensity (A.U.) of Arp2/3 indicates that the levels have increased in the presence of monomer plus ALA, however ALA control also showed increase in the intensity of Arp2/3 (*p* < 0.001) (Fig. 6b). The statistical analysis suggests the significant difference between control (untreated) and Tau and ALA treated groups. To further understand the colocalization index between Arp2/3 and F-actin, the Pearson’s coefficient for colocalization was calculated using ROI, and the analysis indicated an increased degree of colocalization in test groups treated with Tau monomer, aggregates along with ALA (*p* < 0.001) (Fig. 6c). The statistical analysis suggests that the Tau and ALA treated groups are significant with respect to control groups (untreated and ALA). The protein expression of Arp2/3 was also estimated by western blot to understand the changes in expression pattern or the protein density per area. The western blot analysis indicated the increased levels of Arp2/3 in test groups treated with Tau monomer, aggregates along with ALA (Fig. 6d). The quantication of intensity of protein bands normalized to loading control (β-tubulin) was found to increased over Tau and ALA exposure (Fig. 6e). The statistical analysis suggests that the Tau and ALA treated groups are statistically significant with respect to control (untreated) group. The overall results suggest that ALA can involve in the activation of Arp2/3 to induce actin polymerization.

## Discussion

**Fig. 7:**
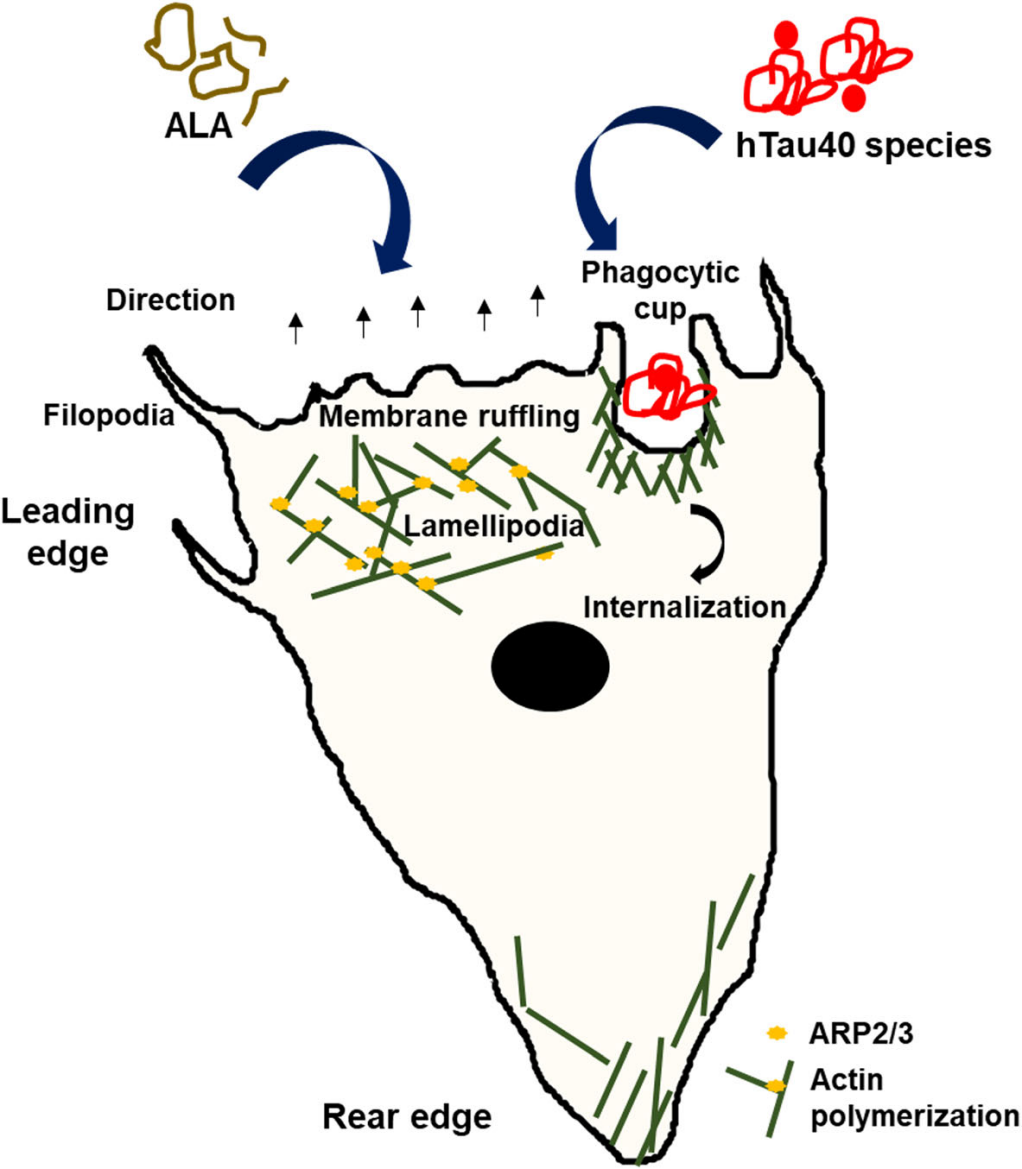
α-Linolenic acid as a modulator of actin cytoskeleton for phagocytosis and migration of microglia. The picture representation suggests effects of ALA after exposure to microglial cells in enhancing extracellular Tau phagocytosis and associated actin-remodeling. ALA enhances phagocytosis of microglia with increased actin dynamics, the actin structure lamellipodia, and filopodia for the migration and promotes membrane ruffling to support the process of phagocytosis

Extracellular deposits of Aβ and intracellular hyperphosphorylated neurofibrillary tangles of Tau depict the hallmark of AD. Accumulation of deposits of Aβ and aggregated Tau seeds in the brain environment prompt cognitive decline and neuronal loss [44]. Microglia as brain macrophages screen the brain environment by constantly elongated processes in the resting stage. After encountering the site of injury or protein deposits, they extend the processes and migrate towards the recognized site, ramified in shape and exert an immune response. In AD, microglia surrounds the Aβ plaques, activates and produce inflammatory response [45]. The gathered microglia towards senile plaques induce the formation of Aβ deposits rather than clearance due to excessive inflammatory cytokines such as IFN-γ and TNF-α [22]. Iba-1 positive microglia also found to accumulate near NFT bearing neurons and induce inflammatory phase [46]. Tau seeds have the ability to propagate in a “prion-like” manner and efficiently alter microglial activation to fabricate inflammatory response [5, 47, 48]. In this condition, inducing anti-inflammatory phenotype of microglia could act as therapeutic strategy and omega-3 fatty acids speed up the process [26]. Omega-3 PUFAs on dietary intake improves cognitive impairment, apoptosis of neurons and inflammatory phase by microglia [25]. DHA and EPA exert polarization of M2 phenotype of microglia along with the excessive expression of phagocytic receptors such as CD206 and anti-inflammatory cytokine IL-4, IL-10 etc. [29, 49]. The cytoskeleton changes needed for the phagocytosis by microglia is also induced *via* omega-3 PUFAs by various mechanism including phosphoinosites signaling [23]. Our results suggest that the necessary actin remodeling for migration and phagocytosis assists the phagocytosis of extracellular Tau species induced by ALA. Co-ordinated polymerization of a branched network of actin filaments is a key phenomenon to generate pushing force for the migratory cells. Extensive motility of immune cells is necessary to locate a target to destroy. The identification of targets *via* receptors initiates a signaling cascade involving actin polymerization to produce membrane protrusions around the target to engulf. After engulfment, the target is enclosed in endocytic vesicle and pinched out from the cell membrane. In this process, actin remodeling plays a major role to induce changes in the plasma membrane, membrane ruffling and protrusions that speed-up phagocytosis [19, 50]. Membrane ruffling shows the involvement of Iba-1 along with actin, whereas Iba-1 mutants hamper membrane ruffling and phagocytic cup formation [20]. The role of Iba-1 as an actin-binding protein has been studied. The activated microglia after 24 h of treatment of ALA and Tau showed high colocalization between actin and Iba-1 at the site of protrusive membrane and enhanced membrane ruffling, lamellipodia and filopodia. ALA was found to increase membrane ruffles to a great extent, which would be effective for increased phagocytosis. Previous studies have been reported for the physical binding of Iba-1 to F-actin, and their involvement in membrane ruffling supports the increased colocalization of Iba-1 and actin in ALA treated cells. These results suggest that the importance of ALA to enhance microglial activation in the presence of Tau species. The activated microglia in the presence of Tau species and ALA might suggest the involvement of ALA as an inducer of phagocytosis to clear the extracellular debris. After CNS injury, microglia has tendency to migrate towards the site of injury and further the migration is supported by rearrangement of actin cytoskeleton [51]. The actin structures and dynamics support migration, whereas lamellipodia structure produces forward protrusive forces and thin protrusion of filopodia inspect distant targets. The ALA treated cells showed greater polarized morphology with presence of dense lammellum at leading edges and uropods at rear edges. The lammellum and filopodia extensions were high in ALA treated cells, which suggests a high directional migration and supports protrusive and contractile migration as microglia sense the extracellular Tau. In previous studies, with the alternative activation of microglia by IL-4 treatment, lammellum with more membrane ruffles have been observed [18]. Similar morphology was observed with ALA treated cells. The actin filament polymerization required for lamellipodia formation is nucleated by Arp2/3 complex. Arp2/3 introduces branching of mother actin filaments to produce a dense branching network of the actin. The density of Arp2/3 in migratory cells is higher at leading edge to produce dense actin network and fade out in other cell body [19, 50]. The increased colocalization between F-actin and Arp2/3 at the leading edge, might suggest the importance of ALA as an inducer of actin polymerization necessary for migration to reach out to extracellular debris. ALA found to enhance Arp2/3 at the leading edge, since it enhances lamellipodia formation. This depicts that ALA has tendency to remodulate actin network (Fig. 7).

## Conclusions

The necessary actin-remodeling for migration and phagocytosis to grasp the target and neutralizing it is one of the therapeutic approach to increase the phagocytic efficiency of microglia. ALA effectively improves the actin remodeling by enhancing lamellipodia, filopodia as well as membrane ruffling. Arp2/3 complex, a nucleation factor for actin polymerization is shown to colocalize with F-actin, which help to improve F-actin turnover. Improved actin remodeling in the presence of ALA may increase the chances of microglia to target the extracellular burden of Tau. Therefore, including ALA as a dietary supplement might be a potential therapeutic approach to clear Tau seeding.

## Materials and methods

### Chemicals and primary antibodies

Luria-Bertani broth (Himedia); Ampicillin, NaCl, Phenylmethylsulfonylfluoride (PMSF), MgCl2, APS, DMSO, Ethanol (Mol Bio grade), Isopropanol (Mol Bio grade) and methanol (Mol Bio grade) were purchased from MP biomedicals; IPTG and Dithiothreitol (DTT) from Calbiochem; MES, BES, SDS, α-Linolenic acid (ALA) (L2376) from Sigma; EGTA, Protease inhibitor cocktail, Tris base, 40% Acrylamide, TEMED from Invitrogen. For cell culture studies, the N9 microglial cell line no. is CVCL- 0452, Roswell Park Memorial Institute (RPMI), Fetal Bovine Serum (FBS), Horse serum, Phosphate buffer saline (PBS, cell biology grade), Trypsin-EDTA, Penicillin-streptomycin, RIPA buffer were also purchased from Invitrogen. MTT reagent and Triton X-100, Trypan-Blue were purchased from Sigma. The coverslip of 12 mm and 18 mm was purchased from Bluestar for immunofluorescence and copper-coated carbon grids for TEM analysis were purchased from Ted Pella, Inc. In immunofluorescence and western blot study we used the following antibodies: β-actin (Thermofisher cat no. MA515739), ARP2 MONO (Thermofisher cat no- 703394), Anti-Iba-1 (Thermo cat no-PA527436**)**, Alexa Fluor-488 Phallodin (A12379), anti-mouse secondary antibody conjugated with Alexa Fluor-488 (Invitrogen, cat no A-11001), Goat anti-Rabbit IgG (H + L) Cross-Adsorbed Secondary Antibody with Alexa Fluor 555 (A-21428), GOXMS ALEXA FLOUR 488 goat anti rabbit (Thermofisher cat no A28175) DAPI (Invitrogen), Goat Anti Mouse secondary antibody Peroxidase conjugated (Thermo fisher 32,430), Prolong Diamond antifade (Thermofisher cat no- P36961).

### Protein expression and purification

Full-length wild type Tau protein (hTau40^wt^) was expressed in BL21* cells with 100 μg/ml of ampicillin antibiotic selection and purified with two-step chromatography methods, cation-exchange chromatography and size-exclusion chromatography [52]. Induction was carried out with 0.5 mM IPTG for 3 h at 37°. In brief, the cell lysate was subjected to 90 °C heating and supernatant was centrifugation at 40000 rpm for 45 min followed by dialysis overnight at 4 °C in 20 mM MES buffer. hTau40 purified by cation-exchange chromatography with Sepharose fast-flow column was used for chromatography. Fractions containing Tau proteins were collected after cation exchange chromatography, it was then concentrated and subjected to size-exclusion chromatography. Size-exclusion chromatography was carried out in the Superdex 75 Hi-load 16/600 column in 1X PBS supplemented with 2 mM DTT. Fractions containing Tau were collected, pooled, concentrated, and the concentration of protein was determined with Bicinchoninic acid (BCA) assay.

### Aggregation assay

Natively unfolded protein Tau undergo aggregation in the presence of poly-anionic agent heparin or arachidonic acid to produce β- sheet structure [35, 53]. Tau aggregation was induced by heparin (MW-17500 Da) in the ratio of 1:4 (heparin to Tau) along with other additives 20 mM BES buffer, 25 mM NaCl, 1 mM DTT, 0.01% NaN_3,_ 20 μl of PIC. Aggregation propensity of Tau was checked with ThS, is a homogeneous mixture of methylation product of dehydrothiotoluidine in sulfonic acid, which can bind to the β-sheet structure. Aggregation kinetics of Tau was studied with 2 μM of Tau and ThS in 1:4 ratios. The excitation wavelength for ThS is 440 nm and the emission wavelength is 521 nm. Three-independent experiments have been performed and further data analysed using Sigmaplot 10.0.

### Transmission electron microscopy

Tau fibrils and ALA vesicles were studied by transmission electron microscopy (TEM) for morphological analysis. 2 μM Tau sample was incubated on 400 mesh, carbon-coated copper grid for observation and stained with 2% Uranyl acetate for the contrast. For ALA vesicles working concentration of 40 μM was taken from the previous studies, for grid preparation [29, 35, 54]. The microscopic observation of Tau fibrils and ALA was carried out by TEM TECNAI T20 120KV.

### Cell culture condition

N9 microglial cells were grown in RPMI media supplemented with 10% heat-inactivated serum, 1% penicillin-streptomycin antibiotic solution and glutamine and grown in T25 flask or 60 mm dish to maintain the culture. Cells were passaged using 0.25% trypsin-EDTA solution after washing with PBS, on attaining 90% confluency or more. For western blot experiment cells were seeded in 6 well plate, while for immunofluorescence experiment cells has been seeded in 12 or 24 well plate on to a coverslip. For α-Linolenic acid preparation, our previously published protocol was followed [29]. Briefly, ALA was dissolved in 100% molecular biology grade ethanol and solubilized at 50 °C in the stock concentration of 20 mM. ALA solution prepared fresh before every experiment. The working concentration of ALA 40 μM was decided according to previous studies, and the final concentration of ethanol in the culture was maintained below 0.5% to avoid solvent-mediated toxicity [29, 35, 54].

### Immunofluorescence analysis

For immunofluorescence 25,000 cells of N9 microglia were seeded on 12 mm coverslip (Bluestar) in 24 well plate supplemented with 10% FBS and 1% penicillin-streptomycin. During treatment of hTau40 monomer/aggregates and ALA the cells were supplemented with 0.5% serum-deprived RPMI media. The treatment of hTau40 monomer, aggregates, ALA was given for 24 h. Cells were then fixed with 4% paraformaldehyde solution for 20 min at room temperature then washed with 1X PBS thrice. Permeabilisation before staining was carried out using 0.2% Triton X-100 for 15 min followed by washing three times with 1X PBS and blocking with 2% serum in 1X PBS for 1 h at room temperature. Primary antibody/phalloidin solution treatment was given to cells overnight at 4 °C in 2% serum in 1X PBS in a moist chamber. The next day, cells were washed with 1X PBS thrice. Then incubated with secondary antibody in 2% serum at 37 °C for 1 h. Further cells were washed with 1X PBS 3 times and counterstained with DAPI (300 nM). Mounting of coverslip was done in Prolong Diamond antifade mounting solution. Images were observed under a 63x oil immersion in Axio observer 7.0 Apotome 2.0 Carl Zeiss microscope.

### Confocal- super-resolution microscopy analysis

To study the actin structures associated with migration, phagocytosis in the presence of ALA, Zeiss LSM 980 with Airy scan 2 in super-resolution mode was used. The immunofluorescence staining for the previously described conditions was carried out with β-Actin (1:500) and Iba-1 (1:500) proteins to study the microglia activation and actin structures. The super-resolution mode helped to resolve and understand the minute cell structures such as lamellipodia, filopodia, membrane ruffling and polarization state of microglia. The image processing was carried out with Zeiss ZEN 2.3 software.

### Western blot

To understand the protein levels in cells, N9 cells (3,00,000 cells/well) were seeded in 6 well plate. The treatment of extracellular Tau monomer, aggregates along with ALA was given to cells for 24 h. Treatment exposure followed by washing with 1X PBS. Cell lysis was carried out using radioimmunoprecipitation (RIPA) assay buffer containing protease inhibitors for 20 min at 4 °C. The cell lysate was centrifuged at 12000 rpm for 20 min. Protein concentration was checked by using Bradford’s assay and equal amount of 75 μg total proteins for all the treatment groups were loaded on polyacrylamide gel electrophoresis of range 4–20% and the gel is electrophoretically transferred to polyvinylidene difluoride membrane and kept for primary antibody β-actin (1:5000), Iba-1 (1:1000), Arp2 mono (1:1000) and β-tubulin (1:2000) binding for overnight at 4 °C. After the incubation blots washed three times with 1X PBST (0.1%: Tween-20). The secondary antibody was incubated for 1 h at RT. Then the membrane was developed using chemiluminescence detection system. The relative quantification of the protein was carried out with loading control β-actin/β-tubulin in all the treatment group.

### Statistical analysis

All the experiment have been performed in three independent biological replicates. The data was plot using SigmaPlot 10.0 and statistical analysis for microscopic images quantification and western blot experiment quantification was done using One-way ANOVA. The statistical significance between different groups has been performed with Tukey Kramer’s post-hoc analysis at 5% level of significance for multiple group comparison. The test groups were compared with control (untreated) as well as with ALA treated group. The data were considered significant if the mean difference between the groups is greater than Tukey’s criteria (X-X’ > T), the *p* value for each group has been calculated and plotted as a star value. The *p* value indication are done as follows ns- non-significant, * indicates *P* ≤ 0.05, ** indicates *P* ≤ 0.01, *** indicates *P* ≤ 0.001. The quantification for microscopic images to understand the absolute intensity of desire protein and the corresponding area of microglia was calculated with Zeiss ZEN 2.3 software for image processing. The colocalization analysis between the two proteins from immunofluorescence images (Pearson’s coefficient) was done using ImageJ software.

## Supplementary Information

**Additional file 1: Figure S1.** α-Linolenic acid enhances membrane ruffling in microglia. **Figure S2.** α-Linolenic acid induces actin polymerization.

## Abbreviations

AD: Alzheimer’s disease
ALA: α- Linolenic acid
Arp2/3: Actin related proteins
CNS: Central nervous system
DHA: Docosahexaenoic acid
EPA: Eicosapentaenoic acid
NFTs: Neurofibrillary tangles
PUFAs: Polyunsaturated fatty acids
