# Supplementary material for: α-Linolenic acid induces clearance of Tau seeds via Actin-remodeling in Microglia
Source: Mol Biomed. 2021 Feb 9;2:4. doi: 10.1186/s43556-021-00028-1 (PMC8607384; doi:10.1186/s43556-021-00028-1)
Supplement: Supplementary file 1 — Additional file 1: Figure S1. α-Linolenic acid enhances membrane ruffling in microglia. Figure S2. α-Linolenic acid induces actin polymerization. [file 43556_2021_28_MOESM1_ESM.pdf]

α-Linolenic acid induces clearance of Tau seeds *via* Actin-remodeling in Microglia

Smita Eknath Desale[1, 2] **Subashchandrabose Chinnathambi**[1, 2, *]

[1]Neurobiology Group, Division of Biochemical Sciences, CSIR-National Chemical Laboratory, Dr. Homi Bhabha Road, Pune, 411008, India

[2]Academy of Scientific and Innovative Research (AcSIR), Ghaziabad, 201002, India

[*]To whom correspondence should be addressed**: Prof. Subashchandrabose Chinnathambi**, Neurobiology group, Division of Biochemical Sciences, CSIR-National Chemical Laboratory (CSIR-NCL), Dr. Homi Bhabha Road, 411008 Pune, India, Telephone: +91-20-25902232, Fax. +91-20-25902648. Email: **s.chinnathambi@ncl.res.in**

Author details

Smita Eknath Desale, email: se.desale@ncl.res.in

Subashchandrabose Chinnathambi, email: s.chinnathambi@ncl.res.in

Supplementary Figure- 1

α-Linolenic acid enhance membrane ruffling in microglia

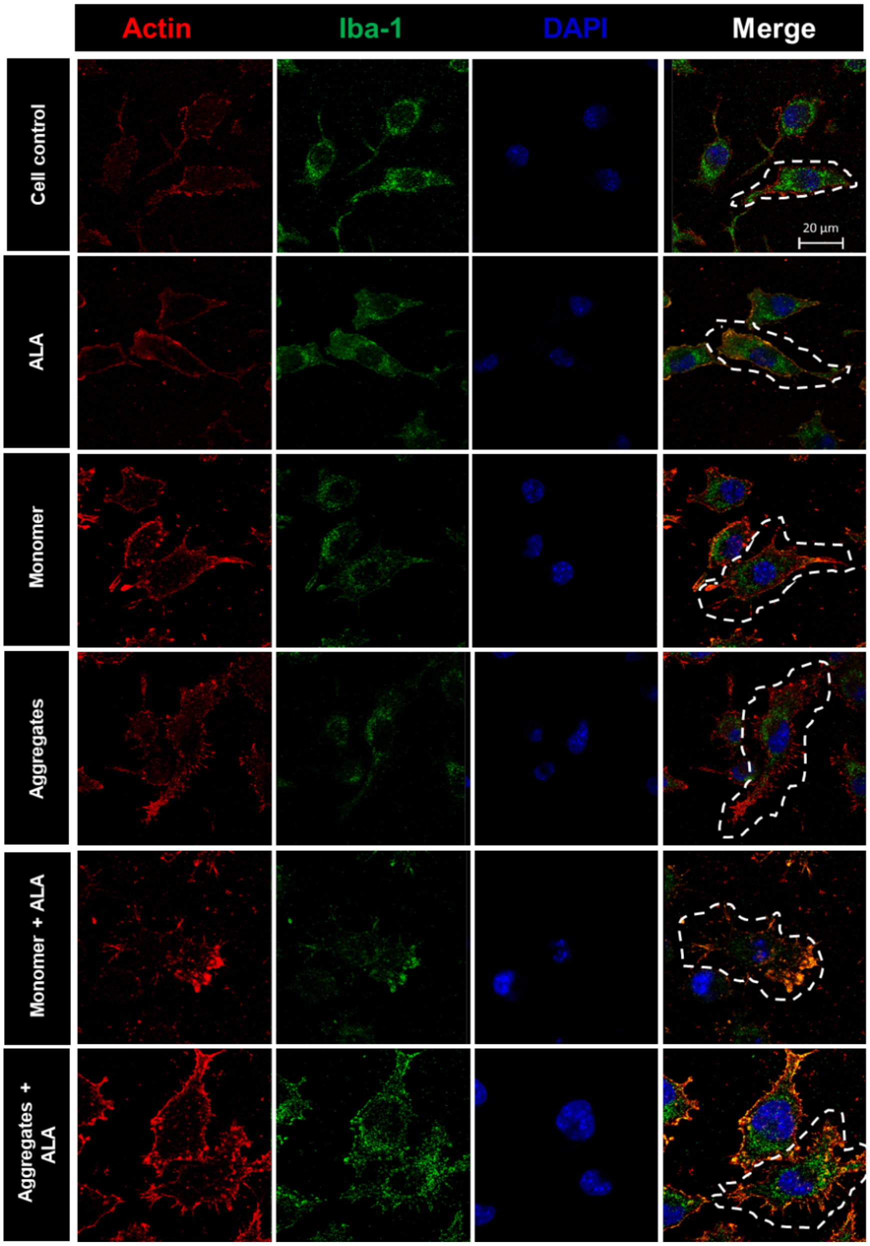

Supplementary figure 1. α-Linolenic acid enhances membrane ruffling in microglia. The image indicates different panel of fluorescence image, scale bar is 20 µm. The enlarged images presented in figure 4 have been taken from marked cells with dotted lines.

Supplementary Figure- 2

α-Linolenic acid induces actin polymerization

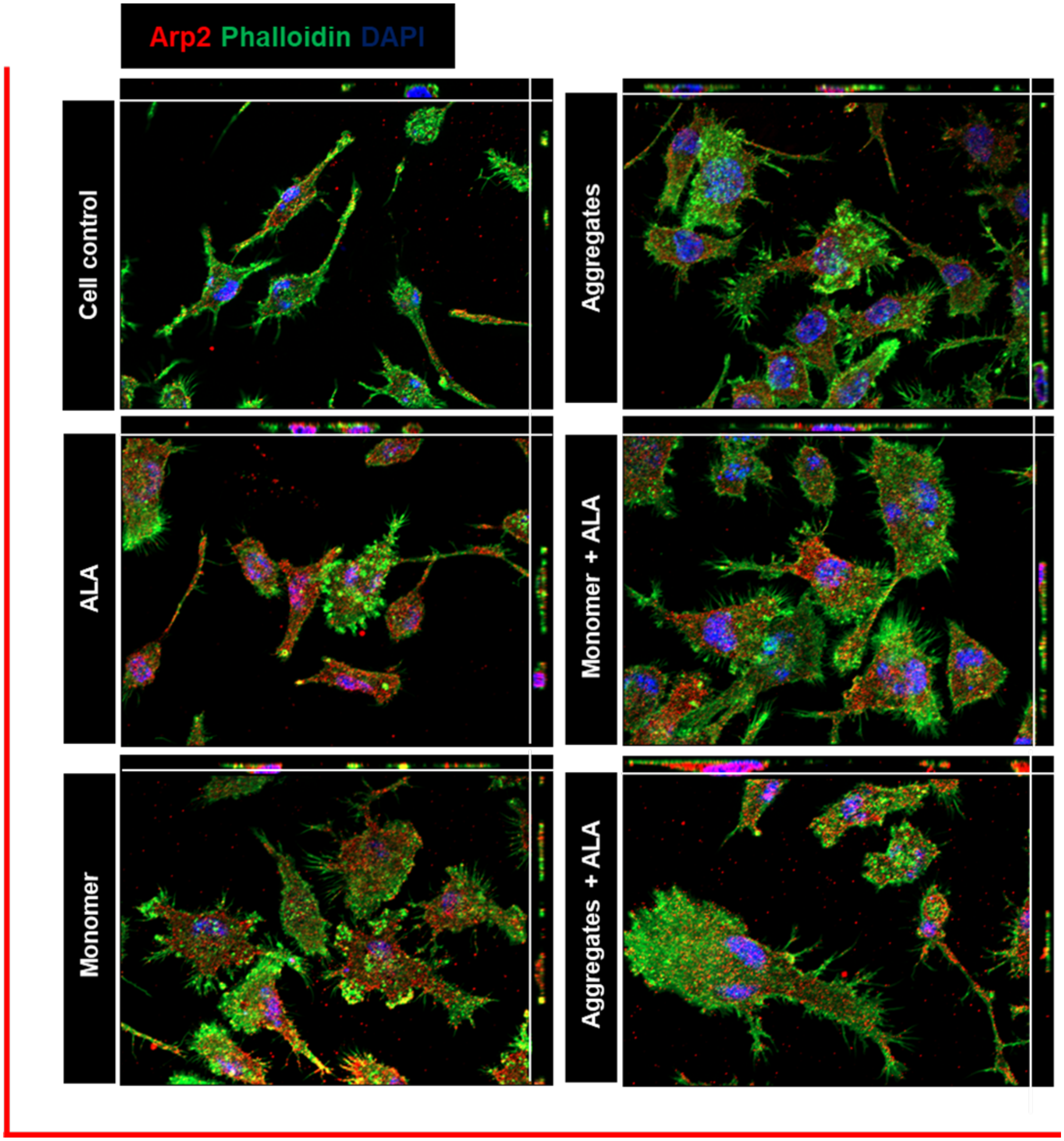

Supplementary figure 2. α-Linolenic acid induces actin polymerization. The orthogonal images indicating XY plane that denotes intracellular colocalization areas of F-actin and Arp2/3 complex.
